# Should Guidelines for Conventional Hemodialysis Initiation in Acute Methanol Poisoning, Be Revised, When no Fomepizloe is Used?

**DOI:** 10.5812/ircmj.3467

**Published:** 2012-11-15

**Authors:** Reza Hekmat, Fariboorz Samini, Bita Dadpour, Faezeh Maghsudloo, Mohammad Javad Mojahedi

**Affiliations:** 1 Department of Nephrology, Ghaem Hospital, Mashhad, Iran

**Keywords:** Hemodialysis, Methanol, Poisoning

## Abstract

**Background:**

Consumption of home-distilled alcohol may lead to epidemic or sporadic cases of severe acute methanol poisoning. The difficulty of establishing strict indications for hemodialysis in acute methanol poisoning is a widely recognized issue.

**Materials and Methods:**

The determination of the clinical, especially hemodialysis, and para clinical factors influencing patient survival in 46 acutely methanol poisoned patients was the aim of this cross sectional retrospective study. Clinical and paraclinical variables compared in surviving and non-surviving patients were hemodialysis and ventilation requirements, the level of consciousness, ABG parameters the serum methanol, creatinine and BUN levels. Only ethanol was used for ADH (Alcohol Dehydrogenize) blockade.

**Results:**

Receiver operative curve characteristics showed that a serum methanol threshold level of 15mg/dl, instead of 25mg/dl, has a better sensitivity and rather the same specificity for predicting patient mortality.

**Conclusions:**

With no fomepizloe and using conventional hemodialysis, lowering the threshold of methanol concentration for hemodialysis initiation, may save lives in acute methanol intoxication.

## 1. Introduction

Consumption of home -distilled alcohol may lead to epidemic or sporadic cases of severe acute methanol poisoning. Significant morbidity and mortality in acute methanol intoxication can be prevented by early diagnosis and treatment (1). While some authors suggest that pre-emptive high flux HD (hemodialysis), be performed in severe methanol poisoning (2), others have claimed that even no hemodialysis may be an option in selected cases, especially when using fomepizole (3), physicians experience or preference has also been mentioned as a criteria for dialysis (4). Even the American Academy of Clinical Toxicology’s practice guidelines are ambiguous with respect to threshold methanol concentration for hemodialysis (5). However, the American Academy of Clinical Toxicology’s practice guidelines suggest that, until more work is done, a methanol concentration of 25 mg/dL remains a reasonable indication for hemodialysis (6). Even according to toxicology textbooks, it is difficult to establish strict indications for hemodialysis in methanol poisoning (7).

## 2. Materials and Methods

Forty six acutely methanol poisoned patients between September 2009 and December 2010 serially entered this cross sectional retrospective study at Imam Reza Hospital, Mashhad, Iran. All patients were hemodialyzed using polysulfane filters and bicarbonate buffer. Patients with severe metabolic acidosis (Ph less than 7.20 refractory to bicarbonate therapy), signs of end organ toxicity (including coma and seizures), a methanol toxic level of more than 25 mg/dl and renal failure (a threefold increase in the serum creatinine or GFR decrease by 75 percent or urine output of < 0.5 mL/kg per hour for 24 hours or anuria for 12 hours) were selected for hemodialysis initiation.4-6 Gas chromatography method was used for measuring the methanol's serum level.

ADH (Alcohol Dehydrogenize) blockade other than ethanol administration (0.8 g/kg loading, then 0.8g/kg /hr until urine methanol positivity), due to unavailability of fomepizole, was not provided. Over all 10 patients in both groups (survived and non-survived) had received hemodialysis.

Independent sample t test and Mann Whitney test were used to compare parameters; serum methanol level, blood PH, blood Co2 pressure and blood bicarbonate level in hemodialyzed, non-hemodialyzed and surviving, non-surviving patients. Regression analysis was used to evaluate the effect of clinical and laboratory parameters on patient survival. The receiver operative curve (ROC) procedure was used to evaluate the specificity and sensitivity of different levels of methanol for predicting patien survival. SPSS package version 15, for statistical analysis, was used. A p-vlue of less than 0.05 was considered significant.

## 3. Results

All four cases of mortality had occurred in hemodialyzed patients. The serum methanol level (Table1), hemodialysis requirement and mechanical ventilation requirement was higher and level of consciousness, was less in non- surviving patients compared to surviving ones.

**Table 1:** Initial serum methanol level, blood PH, blood Co2 pressure and blood bicarbonate level in hemodialyzed, non-hemodialyzed patients and surviving and non-surviving patients.

Factors	Hemodialysis status			Survival status		
	Hemodialyzed Mean ± SD	Non-hemodialyzed Mean ± SD	P-value	Survived Mean+/- Std.	Non-survived Mean ± SD	P-value
**Initial serum methanol level (mg/dl)**	36.30+/-33.34	8.79+/-2.68	0.001	15.53+/-21.95	21.75+/-15.19	0.048
**Initial blood PH**	7.20+/-0.22	7.32+/-0.10	0.03	7.31+/-0.11	7.18+/-0.31	0.11
**Initial blood Co2 pressure ( mm Hg)**	32.02+/-12.99	41.10+/-10.5	0.054	38.76+/-12.07	36.76	0.78
**Initial blood bicarbonate level (m Eq/lit)**	13.07+/-6.90	21.74+/-5.65	0.01	20.68+/-6.81	15.26+/-8.00	0.21
**Initial blood urea nitrogen (mg/dl)**	20.50+/-7.00	16.61+/-6.95	0.21	16.82+/-6.67	23.5	0.19
**Initial serum creatinine (mg/dl)**	1.45+/-0.59	1.30+/-0.71	0.28	1.30+/-0.66	1.5+/-0.42	0.68

There was no difference regarding the sex, age, body weight and height between the patients who survived and those who died (P > 0.05). The mortality rate was 4 (11%). General linear model analysis showed the serum methanol level and hemodialysis requirement as the sole determinants of the patient survival in acute methanol poisoning.

None of the deaths in hemodialyzed patients could be attributed to hemodialysis per se, including catheter insertion complications, cardiac arrhythmia or ion derangements occurring during or immediately after hemodialysis. However the mean duration of hemodialysis in both groups was less than3 hours (mean ± SD = 2.61+/-0.78), although the prescribed duration of hemodialysis for both groups was a minimum of 3 hours. The discrepancy between prescribed and delivered hemodialysis was due to hemodynamic instability during hemodialysis .Hemodynamic instability during different sessions of hemodialysis occurred in both surviving and non-surviving patients by not a different incidence ratio (chi-square P = 0.44). Using receiver operative curves characteristics for finding the threshold of serum methanol concentration , separating surviving from non-surviving patients we found that, the serum methanol threshold level of 25mg/dl, used for initiating hemodialysis, was nearly 100% specific, but only 33% sensitive for predicting mortality. But selecting a serum methanol threshold level of 15mg/dl, instead of more than 25mg/dl, had a significant improving effect on the sensitivity of the mortality prediction, increasing it to 75% while not decreasing the specificity that continued to remain still at 88%.

## 4. Discussion

Non- realization of methanol i.v dose augmentation (150% in the protocol used) during hemodialysis also, the lag of time between the presentation and the beginning of hemodialysis cannot explain the non-survival of the demised patients. Then the other explanation may be that conventional hemodialysis with a short duration time (the mean duration time of the effective hemodialysis in our study was less than 3 hours) may be ineffective or initiated too late for salvaging the patients with severe methanol poisoning, especially when no ADH blockade, other than ethanol is used. The lack of CRRT (continuous renal replacement therapy) facilities may have aggravated this.

Others have also suggested that indications and triage for hemodialysis in methanol poisonings should be modified. A longer duration of hemodialysis to bring the serum methanol levels down to a nontoxic level (8-11), pre-emptive hemodialysis with high-flux membranes (2) or immediate (12) hemodialysis were the options forwarded for this modification. Ghannoum M et al. (9) emphasized that hemodialysis should be considered for all cases of sever methanol intoxication, even when metabolic acidosis is absent, since methanol itself, contributes to the burden of clinical toxicity. Others have given more importance to the blood flow rate of the dialysate, which should be in excess of 250 ml/min, and that bicarbonate bath optimally be enriched with phosphorus and potassium and dialysis session time should be long (2).

Our results reinforce this trend for more intensified and effective hemodialysis in methanol poisoning (12). We conclude that lowering the threshold level for initiating conventional hemodialysis, especially when the antidote fomepizole is not given for any reason, may benefit the patients with acute methanol poisoning.
